# (3*E*,5*E*)-3,5-Bis(4-methyl­benzyl­idene)-1-[3-(piperidin-1-yl)propano­yl]piperidin-4-one

**DOI:** 10.1107/S1600536812031820

**Published:** 2012-07-18

**Authors:** Yalda Kia, Hasnah Osman, Vikneswaran Murugaiyah, Suhana Arshad, Ibrahim Abdul Razak

**Affiliations:** a School of Chemical Sciences, Universiti Sains Malaysia, 11800 USM, Penang, Malaysia; b School of Pharmaceutical Sciences, Universiti Sains Malaysia, 11800 USM, Penang, Malaysia; c School of Physics, Universiti Sains Malaysia, 11800 USM, Penang, Malaysia

## Abstract

In the title compound, C_29_H_34_N_2_O_2_, the central piperidine ring adopts a half-chair conformation, whereas the terminal one adopts a chair conformation. The mean plane of the central piperidine ring [maximum deviation = 0.384 (2) Å] makes dihedral angles of 64.82 (13) and 17.55 (13)° with the benzene rings. In the crystal, mol­ecules are linked into a tape along the *b* axis *via* C—H⋯O inter­actions, generating *R*_2_^2^(20) and *R*_2_^1^(6) graph-set motifs. C—H⋯π inter­actions are observed between the tapes.

## Related literature

For biological activities of α,β-unsaturated ketones, see: Tanaka *et al.* (2003 ▶); Nakayachi *et al.* (2004 ▶); Lee *et al.* (2004 ▶); Hertzberg *et al.* (1989 ▶). For ring conformations, see: Cremer & Pople (1975 ▶). For related structures, see: Aridoss *et al.* (2010 ▶); Kia *et al.* (2011 ▶). For graph-set motifs, see: Bernstein *et al.* (1995 ▶). For the preparation of 1-acryloyl-3,5-dibenzyl­idenepiperidin-4-one, see: Dimmock *et al.* (2001 ▶). For the stability of the temperature controller used for data collection, see: Cosier & Glazer (1986 ▶).
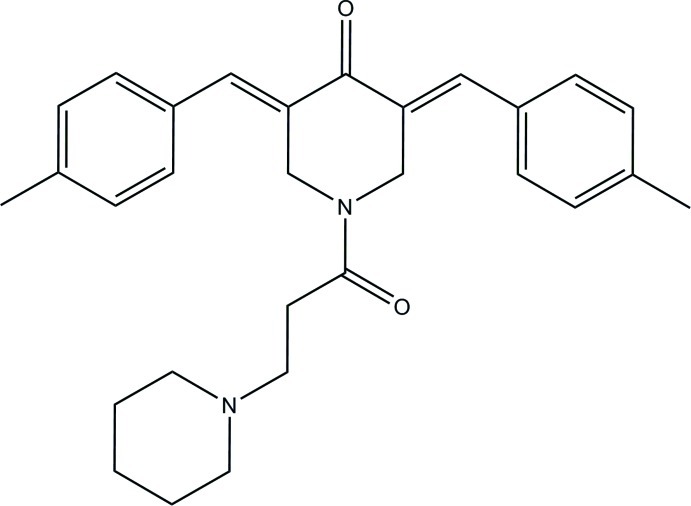


## Experimental

### 

#### Crystal data

C_29_H_34_N_2_O_2_
*M* *_r_* = 442.58
Monoclinic, 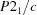

*a* = 12.2913 (8) Å
*b* = 9.9753 (8) Å
*c* = 19.9993 (14) Å
β = 100.884 (4)°
*V* = 2408.0 (3) Å^3^
*Z* = 4
Mo *K*α radiation
μ = 0.08 mm^−1^
*T* = 100 K
0.46 × 0.25 × 0.20 mm

#### Data collection

Bruker SMART APEXII CCD area-detector diffractometer
Absorption correction: multi-scan (*SADABS*; Bruker, 2009 ▶) *T*_min_ = 0.966, *T*_max_ = 0.985
18968 measured reflections
6852 independent reflections
3483 reflections with *I* > *I* > 2σ(*I*)
*R*_int_ = 0.075

#### Refinement

*R*[*F* ^2^ > 2σ(*F* ^2^)] = 0.078
*wR*(*F* ^2^) = 0.228
*S* = 1.04
6852 reflections
300 parameters
H-atom parameters constrained
Δρ_max_ = 0.38 e Å^−3^
Δρ_min_ = −0.37 e Å^−3^

### 

Data collection: *APEX2* (Bruker, 2009 ▶); cell refinement: *SAINT* (Bruker, 2009 ▶); data reduction: *SAINT*; program(s) used to solve structure: *SHELXTL* (Sheldrick, 2008 ▶); program(s) used to refine structure: *SHELXTL*; molecular graphics: *SHELXTL*; software used to prepare material for publication: *SHELXTL* and *PLATON* (Spek, 2009 ▶).

## Supplementary Material

Crystal structure: contains datablock(s) global, I. DOI: 10.1107/S1600536812031820/is5165sup1.cif

Structure factors: contains datablock(s) I. DOI: 10.1107/S1600536812031820/is5165Isup2.hkl

Supplementary material file. DOI: 10.1107/S1600536812031820/is5165Isup3.cml

Additional supplementary materials:  crystallographic information; 3D view; checkCIF report

## Figures and Tables

**Table 1:** Hydrogen-bond geometry (Å, °) *Cg*1 is the centroid of the benzene C14–C19 ring.

*D*—H⋯*A*	*D*—H	H⋯*A*	*D*⋯*A*	*D*—H⋯*A*
C16—H16*A*⋯O2^i^	0.95	2.51	3.346 (3)	147
C21—H21*A*⋯O2^i^	0.98	2.44	3.371 (4)	160
C21—H21*C*⋯O1^ii^	0.98	2.52	3.446 (3)	157
C4—H4*A*⋯*Cg*1^iii^	0.95	2.69	3.526 (3)	148
C27—H27*A*⋯*Cg*1^iv^	0.99	2.74	3.719 (3)	168

Symmetry codes: (i) 
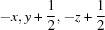
; (ii) 
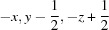
; (iii) 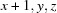; (iv) 
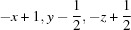
.

**Fig. 1.**
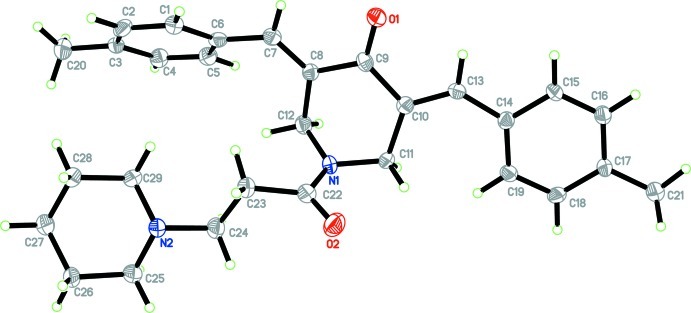
The molecular structure of the title compound, showing 50% probability displacement ellipsoids and the atom-numbering scheme.

**Fig. 2.**
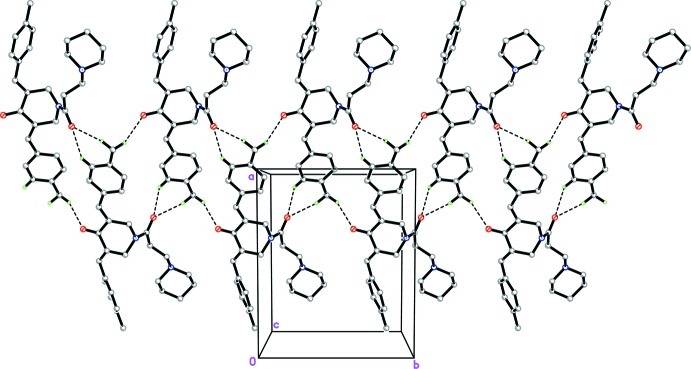
The crystal packing of the title compound. The H atoms not involved in the intermolecular interactions (dashed lines) have been omitted for clarity.

## supplementary crystallographic information

### Comment

Claisen-Schmidt condensation reaction between aldehyde and ketone, leads to biological active class of compound, namely α,β-unsaturated ketones. These compounds show a wide range of biological activities such as enzyme inhibitory (Tanaka *et al.*, 2003), cytotoxic and antitumor (Nakayachi *et al.*, 2004). This conjugated system, O═CH—CH═CH_2_ is the key moiety which promotes the bioactivities in the title compound (Lee *et al.*, 2004). α, β-unsaturated ketones can be considered as a Michael acceptor which is an active moiety showing enzyme inhibitory activity (Hertzberg *et al.*, 1989). Due to these reasons, the crystal structure determination of the title compound was carried out and the results are presented in this paper.

The molecular structure is shown in Fig. 1. The bond lengths and angles are within normal ranges and comparable to the related structures (Aridoss *et al.*, 2010; Kia *et al.*, 2011). The piperidine rings (N1/C8–C12 and N2/C25–C29) adopt different conformations (Cremer & Pople, 1975). N1/C8–C12 adopts a half-chair conformation [puckering parameters, Q= 0.556 (3) Å, Θ= 117.4 (3)° and Φ= 157.4 (3)°], whereas, N2/C25–C29 adopts a chair conformation [puckering parameters, Q= 0.573 (3) Å, Θ= 3.3 (3)° and Φ= 329 (5)°]. The least square plane through both rings form a dihedral angle of 18.29 (13)° between them. The least-square plane of the central piperidine ring [N1/C8–C12, maximum deviation of 0.384 (2) Å at atom N1] forms dihedral angles of 64.82 (13) and 17.55 (13)°, respectively, with the pendant benzene rings (C1–C6 and C14–C19).

In the crystal packing (Fig. 2), molecules are linked into a tape along the *b* axis *via* intermolecular C16—H16A···O2, C21—H21A···O2 and C21—H21C···O1 interactions (Table 1), generating *R*^2^_2_(20) and *R*^1^_2_(6) graph-set motifs (Bernstein *et al.*, 1995). The crystal structure is further stabilized by the intermolecular C4—H4A···*Cg*1 and C27—H27A···*Cg*1 (Table 1) interactions (*Cg*1 is the centroid of the benzene ring, C14–C19).

### Experimental

1-Acryloyl-3,5-dibenzylidenepiperidin-4-one was synthesized as reported in the literature (Dimmock *et al.*, 2001). The title compound was prepared by refluxing 1-acryloyl-3,5-dibenzylidenepiperidin-4-one (0.6 mmol) with piperidine (0.6 mmol) in ethanol. After completion of the reaction as evident from TLC, the mixture was poured into ice. The precipitated solid was filtered and washed with water. The pure solid was then recrystallized from ethanol to afford the title compound as yellow crystals.

### Refinement

All H atoms were positioned geometrically (C—H = 0.95–0.99 Å) and refined using a riding model with *U*_iso_(H) = 1.2 or 1.5*U*_eq_(C). A rotating group model was applied to the methyl groups.

### Crystal data

**Table:**

C_29_H_34_N_2_O_2_	*F*(000) = 952
*M**_r_* = 442.58	*D*_x_ = 1.221 Mg m^−^^3^
Monoclinic, *P*2_1_/*c*	Mo *K*α radiation, λ = 0.71073 Å
Hall symbol: -P 2ybc	Cell parameters from 3337 reflections
*a* = 12.2913 (8) Å	θ = 2.3–29.9°
*b* = 9.9753 (8) Å	µ = 0.08 mm^−^^1^
*c* = 19.9993 (14) Å	*T* = 100 K
β = 100.884 (4)°	Block, yellow
*V* = 2408.0 (3) Å^3^	0.46 × 0.25 × 0.20 mm
*Z* = 4	

### Data collection

**Table:**

Bruker SMART APEXII CCD area-detector diffractometer	6852 independent reflections
Radiation source: fine-focus sealed tube	3483 reflections with *I* > *I* > 2σ(*I*)
Graphite monochromator	*R*_int_ = 0.075
φ and ω scans	θ_max_ = 30.0°, θ_min_ = 1.7°
Absorption correction: multi-scan (*SADABS*; Bruker, 2009)	*h* = −17→17
*T*_min_ = 0.966, *T*_max_ = 0.985	*k* = −13→11
18968 measured reflections	*l* = −26→28

### Refinement

**Table:**

Refinement on *F*^2^	Primary atom site location: structure-invariant direct methods
Least-squares matrix: full	Secondary atom site location: difference Fourier map
*R*[*F*^2^ > 2σ(*F*^2^)] = 0.078	Hydrogen site location: inferred from neighbouring sites
*wR*(*F*^2^) = 0.228	H-atom parameters constrained
*S* = 1.04	*w* = 1/[σ^2^(*F*_o_^2^) + (0.097*P*)^2^ + 0.6841*P*] where *P* = (*F*_o_^2^ + 2*F*_c_^2^)/3
6852 reflections	(Δ/σ)_max_ < 0.001
300 parameters	Δρ_max_ = 0.38 e Å^−^^3^
0 restraints	Δρ_min_ = −0.37 e Å^−^^3^

### Special details

**Table:**

Experimental. The crystal was placed in the cold stream of an Oxford Cryosystems Cobra open-flow nitrogen cryostat (Cosier & Glazer, 1986) operating at 100.0 (1) K.
Geometry. All e.s.d.'s (except the e.s.d. in the dihedral angle between two l.s. planes) are estimated using the full covariance matrix. The cell e.s.d.'s are taken into account individually in the estimation of e.s.d.'s in distances, angles and torsion angles; correlations between e.s.d.'s in cell parameters are only used when they are defined by crystal symmetry. An approximate (isotropic) treatment of cell e.s.d.'s is used for estimating e.s.d.'s involving l.s. planes.
Refinement. Refinement of *F*^2^ against ALL reflections. The weighted *R*-factor *wR* and goodness of fit *S* are based on *F*^2^, conventional *R*-factors *R* are based on *F*, with *F* set to zero for negative *F*^2^. The threshold expression of *F*^2^ > σ(*F*^2^) is used only for calculating *R*-factors(gt) *etc*. and is not relevant to the choice of reflections for refinement. *R*-factors based on *F*^2^ are statistically about twice as large as those based on *F*, and *R*- factors based on ALL data will be even larger.

### Fractional atomic coordinates and isotropic or equivalent isotropic displacement parameters (Å^2^)

**Table:**

	*x*	*y*	*z*	*U*_iso_*/*U*_eq_	
O1	0.33838 (15)	0.3747 (2)	0.18190 (10)	0.0257 (5)	
O2	0.26832 (17)	−0.1316 (2)	0.11812 (11)	0.0389 (6)	
N1	0.39181 (18)	−0.0171 (2)	0.19541 (11)	0.0226 (5)	
N2	0.58294 (18)	−0.2400 (2)	0.05042 (11)	0.0229 (5)	
C1	0.6704 (2)	0.2560 (3)	0.07841 (14)	0.0243 (6)	
H1A	0.6163	0.2964	0.0442	0.029*	
C2	0.7717 (2)	0.2198 (3)	0.06313 (15)	0.0249 (6)	
H2A	0.7854	0.2341	0.0185	0.030*	
C3	0.8540 (2)	0.1623 (3)	0.11272 (15)	0.0236 (6)	
C4	0.8308 (2)	0.1435 (3)	0.17715 (14)	0.0246 (6)	
H4A	0.8863	0.1065	0.2118	0.030*	
C5	0.7293 (2)	0.1768 (3)	0.19258 (14)	0.0243 (6)	
H5A	0.7155	0.1608	0.2371	0.029*	
C6	0.6463 (2)	0.2343 (3)	0.14288 (13)	0.0205 (6)	
C7	0.5366 (2)	0.2719 (3)	0.15616 (13)	0.0215 (6)	
H7A	0.5085	0.3562	0.1387	0.026*	
C8	0.4717 (2)	0.2015 (3)	0.18984 (13)	0.0192 (6)	
C9	0.3625 (2)	0.2580 (3)	0.19813 (13)	0.0199 (6)	
C10	0.2861 (2)	0.1705 (3)	0.22883 (13)	0.0191 (6)	
C11	0.3075 (2)	0.0209 (3)	0.23378 (14)	0.0222 (6)	
H11A	0.3320	−0.0046	0.2821	0.027*	
H11B	0.2380	−0.0279	0.2158	0.027*	
C12	0.4938 (2)	0.0595 (3)	0.21510 (14)	0.0234 (6)	
H12A	0.5536	0.0192	0.1947	0.028*	
H12B	0.5174	0.0590	0.2652	0.028*	
C13	0.2031 (2)	0.2322 (3)	0.25215 (13)	0.0203 (6)	
H13A	0.1985	0.3258	0.2431	0.024*	
C14	0.1187 (2)	0.1830 (3)	0.28866 (13)	0.0199 (6)	
C15	0.0454 (2)	0.2783 (3)	0.30679 (14)	0.0226 (6)	
H15A	0.0513	0.3689	0.2933	0.027*	
C16	−0.0347 (2)	0.2446 (3)	0.34341 (14)	0.0225 (6)	
H16A	−0.0828	0.3120	0.3547	0.027*	
C17	−0.0461 (2)	0.1121 (3)	0.36419 (14)	0.0207 (6)	
C18	0.0264 (2)	0.0167 (3)	0.34587 (14)	0.0234 (6)	
H18A	0.0196	−0.0739	0.3590	0.028*	
C19	0.1072 (2)	0.0493 (3)	0.30944 (13)	0.0220 (6)	
H19A	0.1552	−0.0182	0.2983	0.026*	
C20	0.9643 (2)	0.1219 (3)	0.09596 (16)	0.0323 (7)	
H20A	1.0236	0.1417	0.1348	0.049*	
H20B	0.9771	0.1720	0.0560	0.049*	
H20C	0.9637	0.0256	0.0861	0.049*	
C21	−0.1308 (2)	0.0747 (3)	0.40574 (15)	0.0285 (7)	
H21A	−0.1806	0.1507	0.4076	0.043*	
H21B	−0.0935	0.0511	0.4520	0.043*	
H21C	−0.1737	−0.0024	0.3848	0.043*	
C22	0.3612 (2)	−0.0824 (3)	0.13444 (15)	0.0255 (7)	
C23	0.4436 (2)	−0.0929 (3)	0.08775 (14)	0.0260 (7)	
H23A	0.4927	−0.0134	0.0940	0.031*	
H23B	0.4036	−0.0938	0.0399	0.031*	
C24	0.5139 (2)	−0.2201 (3)	0.10200 (14)	0.0262 (7)	
H24A	0.5620	−0.2133	0.1475	0.031*	
H24B	0.4646	−0.2986	0.1022	0.031*	
C25	0.6299 (2)	−0.3755 (3)	0.05634 (14)	0.0249 (6)	
H25A	0.5691	−0.4418	0.0525	0.030*	
H25B	0.6777	−0.3862	0.1017	0.030*	
C26	0.6971 (2)	−0.4027 (3)	0.00178 (15)	0.0262 (7)	
H26A	0.6476	−0.4022	−0.0434	0.031*	
H26B	0.7315	−0.4925	0.0091	0.031*	
C27	0.7874 (2)	−0.2969 (3)	0.00328 (16)	0.0292 (7)	
H27A	0.8437	−0.3069	0.0455	0.035*	
H27B	0.8245	−0.3100	−0.0361	0.035*	
C28	0.7380 (2)	−0.1587 (3)	0.00057 (16)	0.0292 (7)	
H28A	0.6891	−0.1447	−0.0443	0.035*	
H28B	0.7979	−0.0911	0.0057	0.035*	
C29	0.6719 (2)	−0.1403 (3)	0.05646 (15)	0.0255 (6)	
H29A	0.7215	−0.1495	0.1013	0.031*	
H29B	0.6396	−0.0491	0.0536	0.031*	

### Atomic displacement parameters (Å^2^)

**Table:**

	*U*^11^	*U*^22^	*U*^33^	*U*^12^	*U*^13^	*U*^23^
O1	0.0260 (10)	0.0216 (11)	0.0307 (11)	0.0049 (9)	0.0085 (9)	0.0044 (9)
O2	0.0363 (12)	0.0433 (15)	0.0414 (13)	−0.0166 (11)	0.0183 (11)	−0.0151 (11)
N1	0.0239 (11)	0.0220 (13)	0.0249 (12)	0.0008 (10)	0.0120 (10)	−0.0035 (10)
N2	0.0246 (11)	0.0216 (14)	0.0245 (12)	0.0006 (10)	0.0098 (10)	−0.0003 (10)
C1	0.0259 (14)	0.0223 (16)	0.0255 (14)	0.0006 (12)	0.0070 (12)	0.0005 (12)
C2	0.0298 (15)	0.0238 (16)	0.0232 (14)	−0.0056 (12)	0.0101 (12)	0.0028 (12)
C3	0.0223 (13)	0.0180 (15)	0.0325 (16)	−0.0036 (11)	0.0103 (12)	−0.0052 (12)
C4	0.0192 (13)	0.0281 (17)	0.0256 (15)	−0.0023 (12)	0.0019 (11)	−0.0021 (13)
C5	0.0292 (15)	0.0244 (16)	0.0194 (14)	−0.0030 (12)	0.0044 (12)	−0.0027 (12)
C6	0.0225 (13)	0.0160 (14)	0.0228 (14)	−0.0038 (11)	0.0036 (11)	−0.0015 (11)
C7	0.0226 (13)	0.0195 (15)	0.0220 (14)	−0.0009 (11)	0.0033 (11)	−0.0020 (11)
C8	0.0197 (13)	0.0198 (15)	0.0185 (13)	−0.0001 (11)	0.0047 (11)	−0.0007 (11)
C9	0.0219 (13)	0.0228 (16)	0.0149 (12)	0.0025 (11)	0.0027 (10)	−0.0031 (11)
C10	0.0204 (13)	0.0211 (15)	0.0156 (13)	−0.0008 (11)	0.0033 (10)	−0.0004 (11)
C11	0.0264 (14)	0.0184 (15)	0.0252 (14)	−0.0029 (12)	0.0137 (12)	−0.0001 (12)
C12	0.0221 (13)	0.0235 (16)	0.0253 (15)	0.0031 (11)	0.0064 (12)	0.0009 (12)
C13	0.0219 (13)	0.0161 (14)	0.0231 (14)	−0.0004 (11)	0.0043 (11)	−0.0015 (11)
C14	0.0176 (12)	0.0221 (15)	0.0203 (13)	−0.0016 (11)	0.0041 (11)	−0.0027 (11)
C15	0.0229 (13)	0.0172 (15)	0.0292 (15)	0.0047 (11)	0.0085 (12)	0.0018 (12)
C16	0.0224 (13)	0.0226 (16)	0.0240 (14)	0.0023 (12)	0.0078 (11)	0.0003 (12)
C17	0.0200 (13)	0.0217 (16)	0.0219 (14)	0.0011 (11)	0.0075 (11)	−0.0023 (12)
C18	0.0280 (14)	0.0188 (15)	0.0250 (14)	−0.0031 (12)	0.0093 (12)	0.0021 (12)
C19	0.0267 (14)	0.0183 (15)	0.0223 (14)	0.0002 (11)	0.0080 (12)	−0.0018 (11)
C20	0.0296 (15)	0.0337 (19)	0.0368 (18)	−0.0018 (14)	0.0143 (14)	−0.0030 (15)
C21	0.0287 (15)	0.0249 (17)	0.0342 (17)	0.0016 (13)	0.0121 (13)	−0.0002 (13)
C22	0.0286 (15)	0.0220 (16)	0.0280 (15)	−0.0002 (12)	0.0105 (12)	−0.0013 (13)
C23	0.0286 (14)	0.0266 (17)	0.0253 (15)	−0.0003 (13)	0.0113 (12)	−0.0013 (13)
C24	0.0287 (15)	0.0274 (17)	0.0247 (15)	0.0014 (13)	0.0108 (12)	0.0014 (13)
C25	0.0283 (14)	0.0202 (16)	0.0274 (15)	0.0015 (12)	0.0082 (12)	0.0013 (12)
C26	0.0292 (15)	0.0232 (16)	0.0296 (15)	0.0041 (12)	0.0142 (13)	0.0011 (13)
C27	0.0277 (15)	0.0261 (17)	0.0368 (17)	0.0033 (13)	0.0135 (13)	−0.0010 (14)
C28	0.0301 (15)	0.0251 (17)	0.0355 (17)	−0.0019 (13)	0.0143 (14)	0.0001 (14)
C29	0.0295 (14)	0.0208 (16)	0.0284 (15)	−0.0047 (12)	0.0116 (13)	−0.0009 (12)

### Geometric parameters (Å, º)

**Table:**

O1—C9	1.230 (3)	C15—C16	1.375 (4)
O2—C22	1.229 (3)	C15—H15A	0.9500
N1—C22	1.371 (4)	C16—C17	1.400 (4)
N1—C11	1.451 (3)	C16—H16A	0.9500
N1—C12	1.457 (3)	C17—C18	1.399 (4)
N2—C25	1.465 (4)	C17—C21	1.496 (4)
N2—C29	1.466 (3)	C18—C19	1.377 (4)
N2—C24	1.468 (4)	C18—H18A	0.9500
C1—C2	1.384 (4)	C19—H19A	0.9500
C1—C6	1.394 (4)	C20—H20A	0.9800
C1—H1A	0.9500	C20—H20B	0.9800
C2—C3	1.399 (4)	C20—H20C	0.9800
C2—H2A	0.9500	C21—H21A	0.9800
C3—C4	1.384 (4)	C21—H21B	0.9800
C3—C20	1.511 (4)	C21—H21C	0.9800
C4—C5	1.381 (4)	C22—C23	1.505 (4)
C4—H4A	0.9500	C23—C24	1.532 (4)
C5—C6	1.405 (4)	C23—H23A	0.9900
C5—H5A	0.9500	C23—H23B	0.9900
C6—C7	1.471 (4)	C24—H24A	0.9900
C7—C8	1.336 (4)	C24—H24B	0.9900
C7—H7A	0.9500	C25—C26	1.513 (4)
C8—C9	1.493 (4)	C25—H25A	0.9900
C8—C12	1.511 (4)	C25—H25B	0.9900
C9—C10	1.497 (4)	C26—C27	1.527 (4)
C10—C13	1.349 (4)	C26—H26A	0.9900
C10—C11	1.515 (4)	C26—H26B	0.9900
C11—H11A	0.9900	C27—C28	1.504 (4)
C11—H11B	0.9900	C27—H27A	0.9900
C12—H12A	0.9900	C27—H27B	0.9900
C12—H12B	0.9900	C28—C29	1.512 (4)
C13—C14	1.462 (4)	C28—H28A	0.9900
C13—H13A	0.9500	C28—H28B	0.9900
C14—C15	1.403 (4)	C29—H29A	0.9900
C14—C19	1.412 (4)	C29—H29B	0.9900
			
C22—N1—C11	119.4 (2)	C19—C18—C17	122.5 (3)
C22—N1—C12	124.6 (2)	C19—C18—H18A	118.8
C11—N1—C12	112.6 (2)	C17—C18—H18A	118.8
C25—N2—C29	110.1 (2)	C18—C19—C14	120.1 (3)
C25—N2—C24	109.8 (2)	C18—C19—H19A	120.0
C29—N2—C24	111.8 (2)	C14—C19—H19A	120.0
C2—C1—C6	121.4 (3)	C3—C20—H20A	109.5
C2—C1—H1A	119.3	C3—C20—H20B	109.5
C6—C1—H1A	119.3	H20A—C20—H20B	109.5
C1—C2—C3	120.7 (3)	C3—C20—H20C	109.5
C1—C2—H2A	119.7	H20A—C20—H20C	109.5
C3—C2—H2A	119.7	H20B—C20—H20C	109.5
C4—C3—C2	117.8 (2)	C17—C21—H21A	109.5
C4—C3—C20	121.4 (3)	C17—C21—H21B	109.5
C2—C3—C20	120.7 (3)	H21A—C21—H21B	109.5
C5—C4—C3	121.9 (3)	C17—C21—H21C	109.5
C5—C4—H4A	119.0	H21A—C21—H21C	109.5
C3—C4—H4A	119.0	H21B—C21—H21C	109.5
C4—C5—C6	120.4 (3)	O2—C22—N1	120.8 (3)
C4—C5—H5A	119.8	O2—C22—C23	120.5 (3)
C6—C5—H5A	119.8	N1—C22—C23	118.7 (2)
C1—C6—C5	117.7 (2)	C22—C23—C24	111.3 (2)
C1—C6—C7	119.4 (2)	C22—C23—H23A	109.4
C5—C6—C7	123.0 (3)	C24—C23—H23A	109.4
C8—C7—C6	127.7 (3)	C22—C23—H23B	109.4
C8—C7—H7A	116.1	C24—C23—H23B	109.4
C6—C7—H7A	116.1	H23A—C23—H23B	108.0
C7—C8—C9	119.5 (2)	N2—C24—C23	111.2 (2)
C7—C8—C12	125.1 (2)	N2—C24—H24A	109.4
C9—C8—C12	115.2 (2)	C23—C24—H24A	109.4
O1—C9—C8	120.4 (3)	N2—C24—H24B	109.4
O1—C9—C10	121.5 (2)	C23—C24—H24B	109.4
C8—C9—C10	118.1 (2)	H24A—C24—H24B	108.0
C13—C10—C9	116.7 (3)	N2—C25—C26	111.6 (2)
C13—C10—C11	124.1 (2)	N2—C25—H25A	109.3
C9—C10—C11	119.2 (2)	C26—C25—H25A	109.3
N1—C11—C10	110.8 (2)	N2—C25—H25B	109.3
N1—C11—H11A	109.5	C26—C25—H25B	109.3
C10—C11—H11A	109.5	H25A—C25—H25B	108.0
N1—C11—H11B	109.5	C25—C26—C27	110.8 (2)
C10—C11—H11B	109.5	C25—C26—H26A	109.5
H11A—C11—H11B	108.1	C27—C26—H26A	109.5
N1—C12—C8	108.1 (2)	C25—C26—H26B	109.5
N1—C12—H12A	110.1	C27—C26—H26B	109.5
C8—C12—H12A	110.1	H26A—C26—H26B	108.1
N1—C12—H12B	110.1	C28—C27—C26	110.2 (2)
C8—C12—H12B	110.1	C28—C27—H27A	109.6
H12A—C12—H12B	108.4	C26—C27—H27A	109.6
C10—C13—C14	132.4 (3)	C28—C27—H27B	109.6
C10—C13—H13A	113.8	C26—C27—H27B	109.6
C14—C13—H13A	113.8	H27A—C27—H27B	108.1
C15—C14—C19	117.3 (3)	C27—C28—C29	110.8 (3)
C15—C14—C13	116.9 (3)	C27—C28—H28A	109.5
C19—C14—C13	125.7 (3)	C29—C28—H28A	109.5
C16—C15—C14	122.1 (3)	C27—C28—H28B	109.5
C16—C15—H15A	119.0	C29—C28—H28B	109.5
C14—C15—H15A	119.0	H28A—C28—H28B	108.1
C15—C16—C17	120.7 (3)	N2—C29—C28	110.7 (2)
C15—C16—H16A	119.6	N2—C29—H29A	109.5
C17—C16—H16A	119.6	C28—C29—H29A	109.5
C18—C17—C16	117.4 (3)	N2—C29—H29B	109.5
C18—C17—C21	121.3 (3)	C28—C29—H29B	109.5
C16—C17—C21	121.3 (3)	H29A—C29—H29B	108.1
			
C6—C1—C2—C3	−1.2 (4)	C11—C10—C13—C14	3.1 (4)
C1—C2—C3—C4	−0.2 (4)	C10—C13—C14—C15	178.4 (3)
C1—C2—C3—C20	179.6 (3)	C10—C13—C14—C19	1.0 (5)
C2—C3—C4—C5	1.4 (4)	C19—C14—C15—C16	0.2 (4)
C20—C3—C4—C5	−178.3 (3)	C13—C14—C15—C16	−177.5 (2)
C3—C4—C5—C6	−1.3 (4)	C14—C15—C16—C17	0.0 (4)
C2—C1—C6—C5	1.3 (4)	C15—C16—C17—C18	−0.3 (4)
C2—C1—C6—C7	−178.7 (3)	C15—C16—C17—C21	178.3 (3)
C4—C5—C6—C1	0.0 (4)	C16—C17—C18—C19	0.6 (4)
C4—C5—C6—C7	179.9 (3)	C21—C17—C18—C19	−178.0 (2)
C1—C6—C7—C8	136.4 (3)	C17—C18—C19—C14	−0.5 (4)
C5—C6—C7—C8	−43.5 (4)	C15—C14—C19—C18	0.1 (4)
C6—C7—C8—C9	179.7 (2)	C13—C14—C19—C18	177.6 (2)
C6—C7—C8—C12	−7.0 (4)	C11—N1—C22—O2	−14.0 (4)
C7—C8—C9—O1	−9.2 (4)	C12—N1—C22—O2	−171.4 (3)
C12—C8—C9—O1	176.9 (2)	C11—N1—C22—C23	166.0 (2)
C7—C8—C9—C10	173.0 (2)	C12—N1—C22—C23	8.7 (4)
C12—C8—C9—C10	−1.0 (3)	O2—C22—C23—C24	−89.8 (3)
O1—C9—C10—C13	−14.8 (4)	N1—C22—C23—C24	90.2 (3)
C8—C9—C10—C13	163.1 (2)	C25—N2—C24—C23	−167.5 (2)
O1—C9—C10—C11	166.9 (2)	C29—N2—C24—C23	70.0 (3)
C8—C9—C10—C11	−15.3 (3)	C22—C23—C24—N2	171.9 (2)
C22—N1—C11—C10	−105.7 (3)	C29—N2—C25—C26	−59.3 (3)
C12—N1—C11—C10	54.2 (3)	C24—N2—C25—C26	177.2 (2)
C13—C10—C11—N1	171.5 (2)	N2—C25—C26—C27	55.5 (3)
C9—C10—C11—N1	−10.3 (3)	C25—C26—C27—C28	−52.5 (3)
C22—N1—C12—C8	87.9 (3)	C26—C27—C28—C29	54.1 (3)
C11—N1—C12—C8	−70.9 (3)	C25—N2—C29—C28	60.6 (3)
C7—C8—C12—N1	−132.5 (3)	C24—N2—C29—C28	−177.1 (2)
C9—C8—C12—N1	41.0 (3)	C27—C28—C29—N2	−58.7 (3)
C9—C10—C13—C14	−175.2 (2)		

### Hydrogen-bond geometry (Å, º)

*Cg*1 is the centroid of the benzene C14–C19 ring.

**Table:**

*D*—H···*A*	*D*—H	H···*A*	*D*···*A*	*D*—H···*A*
C16—H16*A*···O2^i^	0.95	2.51	3.346 (3)	147
C21—H21*A*···O2^i^	0.98	2.44	3.371 (4)	160
C21—H21*C*···O1^ii^	0.98	2.52	3.446 (3)	157
C4—H4*A*···*Cg*1^iii^	0.95	2.69	3.526 (3)	148
C27—H27*A*···*Cg*1^iv^	0.99	2.74	3.719 (3)	168

Symmetry codes: (i) −*x*, *y*+1/2, −*z*+1/2; (ii) −*x*, *y*−1/2, −*z*+1/2; (iii) *x*+1, *y*, *z*; (iv) −*x*+1, *y*−1/2, −*z*+1/2.
